# Supplementary material for: Suppression weakens unwanted memories via a sustained reduction of neural reactivation
Source: eLife. 2022 Mar 30;11:e71309. doi: 10.7554/eLife.71309 (PMC8967383; doi:10.7554/eLife.71309)
Supplement: Transparent reporting form [file elife-71309-transrepform1.pdf]

eLife

Westbrook Centre
Milton Road
Cambridge CB4 1YG, UK

P 01223 855340
W elifesciences.org
T @elife

eLife's transparent reporting form

We encourage authors to provide detailed information *within their submission* to facilitate the interpretation and replication of experiments. Authors can upload supporting documentation to indicate the use of appropriate reporting guidelines for health-related research (see EQUATOR Network), life science research (see the BioSharing Information Resource), or the ARRIVE guidelines for reporting work involving animal research. Where applicable, authors should refer to any relevant reporting standards documents in this form.

If you have any questions, please consult our Journal Policies and/or contact us: editorial@elifesciences.org.

Sample-size estimation

- You should state whether an appropriate sample size was computed when the study was being designed
- You should state the statistical method of sample size computation and any required assumptions
- If no explicit power analysis was used, you should describe how you decided what sample (replicate) size (number) to use

Please outline where this information can be found within the submission (e.g., sections or figure legends), or explain why this information doesn't apply to your submission:

> Due to the novelty of the examined effect, we did not perform a power analysis. Instead, we chose our sample size to exceed those in previous pertinent studies on memory suppression (Benoit & Anderson, 2012, Gagnepain et al., 2014) and forgetting (Wimber et al., 2015, Poppenk & Norman, 2014). This information can be found in the Materials and Methods. Our final included sample size exceeded these studies by at least 37.5 %.

Replicates

- You should report how often each experiment was performed
- You should include a definition of biological versus technical replication
- The data obtained should be provided and sufficient information should be provided to indicate the number of independent biological and/or technical replicates
- If you encountered any outliers, you should describe how these were handled
- Criteria for exclusion/inclusion of data should be clearly stated
- High-throughput sequence data should be uploaded before submission, with a private link for reviewers provided (these are available from both GEO and ArrayExpress)

Please outline where this information can be found within the submission (e.g., sections or figure legends), or explain why this information doesn't apply to your submission:

> We replicated the behavioral effect (i.e., the suppression-induced reduction in vividness) in the two studies, both of which are reported in the manuscript. Only one of those was conducted as an MRI study. For that study, data of four participants were excluded as described in the Participants section of the Materials and Methods.

eLife Sciences Publications, Ltd is a limited liability non-profit non-stock corporation incorporated in the State of Delaware, USA, with company number 5030732, and is registered in the UK with company number FC030576 and branch number BR015634 at the address Westbrook Centre, Milton Road, Cambridge CB4 1YG.

Statistical reporting

- Statistical analysis methods should be described and justified
- Raw data should be presented in figures whenever informative to do so (typically when N per group is less than 10)
- For each experiment, you should identify the statistical tests used, exact values of N, definitions of center, methods of multiple test correction, and dispersion and precision measures (e.g., mean, median, SD, SEM, confidence intervals; and, for the major substantive results, a measure of effect size (e.g., Pearson's r, Cohen's d)
- Report exact p-values wherever possible alongside the summary statistics and 95% confidence intervals. These should be reported for all key questions and not only when the p-value is less than 0.05.

Please outline where this information can be found within the submission (e.g., sections or figure legends), or explain why this information doesn't apply to your submission:

Details of statistical tests, mean values, 95% CI, SD or SEM, effect sizes, and exact p-values are clearly reported for all analyses described in the Materials and Methods as well as in the Results sections. The figures depict individual data points plus summary statistics and distributions.

(For large datasets, or papers with a very large number of statistical tests, you may upload a single table file with tests, Ns, etc., with reference to sections in the manuscript.)

Group allocation

- Indicate how samples were allocated into experimental groups (in the case of clinical studies, please specify allocation to treatment method); if randomization was used, please also state if restricted randomization was applied
- Indicate if masking was used during group allocation, data collection and/or data analysis

Please outline where this information can be found within the submission (e.g., sections or figure legends), or explain why this information doesn't apply to your submission:

N/A. We used a within-subjects manipulation so that all participants completed all experimental conditions.

Additional data files ("source data")

- We encourage you to upload relevant additional data files, such as numerical data that are represented as a graph in a figure, or as a summary table
- Where provided, these should be in the most useful format, and they can be uploaded as "Source data" files linked to a main figure or table
- Include model definition files including the full list of parameters used
- Include code used for data analysis (e.g., R, MatLab)
- Avoid stating that data files are "available upon request"

Please indicate the figures or tables for which source data files have been provided:

eLife Sciences Publications, Ltd is a limited liability non-profit non-stock corporation incorporated in the State of Delaware, USA, with company number 5030732, and is registered in the UK with company number FC030576 and branch number BR015634 at the address Westbrook Centre, Milton Road, Cambridge CB4 1YG.

All data are publicly available that support the findings, i.e., that are depicted in the figures and that the inferential statistics are based on (https://osf.io/swxtd/?view_only=27da0e7814d24c3fafecddc2ab0a1163). These are provided as source data files.
We additionally provide the statistical map of the univariate fMRI analysis (https://neurovault.org/collections/KAZGAACE/) as well as all custom code used to analyze the data (including a full R Markdown) (https://osf.io/swxtd/?view_only=27da0e7814d24c3fafecddc2ab0a1163).
However, we can not openly share the raw, unprocessed MRI data, because the participants did not give consent for these data to be released publicly within the General Data Protection Regulation 2016/679 of the EU. The data will be stored on the servers of the Max Planck Institute of Cognitive and Brain Sciences and can be made available to individual researchers on informal request to the corresponding author.

eLife Sciences Publications, Ltd is a limited liability non-profit non-stock corporation incorporated in the State of Delaware, USA, with company number 5030732, and is registered in the UK with company number FC030576 and branch number BR015634 at the address Westbrook Centre, Milton Road, Cambridge CB4 1YG.
